# Cardiometabolic health in adults born with very low birth weight—a sibling study

**DOI:** 10.1038/s41390-023-02828-3

**Published:** 2023-09-27

**Authors:** Nina Kaseva, Juho Kuula, Samuel Sandboge, Helena Hauta-alus, Johan Björkqvist, Petteri Hovi, Johan G. Eriksson, Terhi Vihervaara, Kirsi H. Pietiläinen, Eero Kajantie

**Affiliations:** 1 Finnish Institute for Health and Welfare, Helsinki, Finland; 2 Department of Radiology, HUS Medical Imaging Center, University of Helsinki and Helsinki University Hospital, Helsinki, Finland; 3 Psychology/Welfare Sciences, Faculty of Social Sciences, University of Tampere, Tampere, Finland; 4 Children’s Hospital, and Pediatric Research Center, University of Helsinki and Helsinki University Hospital, Helsinki, Finland; 5 PEDEGO Research Unit, University of Oulu, Oulu, Finland; 6 Research Program for Clinical and Molecular Metabolism (CAMM), Faculty of Medicine, University of Helsinki, Helsinki, Finland; 7 Department of General Practice and Primary Health Care, University of Helsinki and Helsinki University Hospital, Helsinki, Finland; 8 Folkhälsan Research Center, Helsinki, Finland; 9 Department of Obstetrics and Gynecology and Human Translational Research Programme, Yong Loo Lin School of Medicine, National University of Singapore, Singapore, Singapore; 10 Singapore Institute for Clinical Sciences (SICS), Agency for Science, Technology and Research (A∗STAR), Singapore, Singapore; 11 Obesity Research Unit, Research Program for Clinical and Molecular Metabolism, Faculty of Medicine, University of Helsinki, Helsinki, Finland; 12 Obesity Center, Abdominal Center, Helsinki University Hospital and University of Helsinki, Helsinki, Finland; 13 Department of Clinical and Molecular Medicine, Norwegian University of Science and Technology, Trondheim, Norway

## Abstract

**Background:**

Preterm survivors have increased risk for impaired cardiometabolic health. We assessed glucose regulation and cardiometabolic biomarkers in adult very low birth weight (VLBW, <1500 g) survivors, using siblings as controls.

**Methods:**

VLBW-participants were matched with term-born, same-sex siblings. At mean age 29.2 years (SD 3.9), 74 VLBW-adults and 70 siblings underwent a 2-h 75 g oral glucose tolerance test and blood tests for assessment of cardiometabolic biomarkers.

**Results:**

Of participants, 23 (31%) VLBW and 11 (16%) sibling-controls met World Health Organization criteria for impaired glucose regulation (OR adjusted for age and sex 2.5, 95% CI: 1.1 to 5.8).

Adjusting for age and sex, VLBW-participants showed 9.2% higher 2-h glucose (95% CI: 0.4% to 18.8%) than their siblings. Also, fasting (13.4%, −0.3% to 29.0%) and 2-h free fatty acids (15.6%, −2.4% to 36.9%) were higher in VLBW-participants. These differences were statistically significant only after further adjusting for confounders. No statistically significant differences were found regarding other measured biomarkers, including insulin resistance, atherogenic lipid profiles or liver tests.

**Conclusions:**

VLBW-adults showed more impaired fatty acid metabolism and glucose regulation. Differences in cardiometabolic biomarkers were smaller than in previous non-sibling studies. This may partly be explained by shared familial, genetic, or environmental factors.

**Impact:**

- At young adult age, odds for impaired glucose regulation were 3.4-fold in those born at very low birth weight, compared to same-sex term-born siblings.
- Taking into consideration possible unmeasured, shared familial confounders, we compared cardiometabolic markers in adults born preterm at very low birth weight with term-born siblings.
- Prematurity increased risk for impaired glucose regulation, unrelated to current participant characteristics, including body mass index.
- In contrast to previous studies, differences in insulin resistance were not apparent, suggesting that insulin resistance may partially be explained by factors shared between siblings. Also, common cardiometabolic biomarkers were similar within sibling pairs.

## Introduction

Annually 14.8 million babies are born preterm (<37 weeks of gestation), constituting approximately 1/10th of all livebirths.^1^ The survival rate is 98% in high-income countries and 90% globally.^1^ Preterm birth may have life-long consequences on overall health and function including cognitive functioning, attention, neuromotor abilities, physical fitness, lung function, and bone health.^2–7^ Moreover, prematurity is influencing later cardiovascular health and is associated with the components of metabolic syndrome and cardiovascular disease in adult life.^8,9^ Prematurity has, for instance, been linked to insulin resistance, higher fat mass, blood pressure, cholesterol, fasting glucose and insulin levels in comparisons between preterm and term-born adults.^8,10–12^

While many previous studies use carefully selected comparison groups and adjust for important confounders, such studies cannot exclude residual confounding by, for example, unmeasured socio-economic, lifestyle or genetic factors that predispose both to preterm birth and adult cardiovascular outcomes. Efforts have been made to overcome such confounding by utilizing sibling information from large register data. For example, in a retrospective national cohort study of 2.2 million adults, preterm birth was associated with increased risk of lipid disorders in adulthood as compared with full-term birth.^13^ In that study, 84% of the cohort had a sibling, and based on co-sibling analyses, the observed increase in risk of lipid disorders was largely explained by shared genetic or environmental factors in families.^13^ Another family-based study of 386485 singleton-born men, conducted comparisons both between non-siblings and within siblings. The study found inverse associations of birth weight and gestational age with systolic blood pressure at ages 17 to 19 years.^14^ These results were not explained by family socioeconomic position or other factors shared by siblings. In a further Swedish population-based cohort study of 2.1 million participants, which also included co-sibling analyses, preterm birth was associated with an increased risk for ischemic heart disease in adulthood, independently of shared familial genetic or environmental factors.^9^

Despite evident benefits, the sibling study design has rarely been used in clinical birth cohort studies of adults born preterm. We examined associations of preterm birth at very low birth weight (VLBW, birth weight ≤1500 g) with adult cardiometabolic biomarkers using same-sex siblings, born at term, as controls. We hypothesized that VLBW affects cardiometabolic risk factors at adult age, and that possible differences compared with siblings born at term might be less pronounced, considering common genetic and background factors.

## Methods

### Participants

**Fig. 1:**
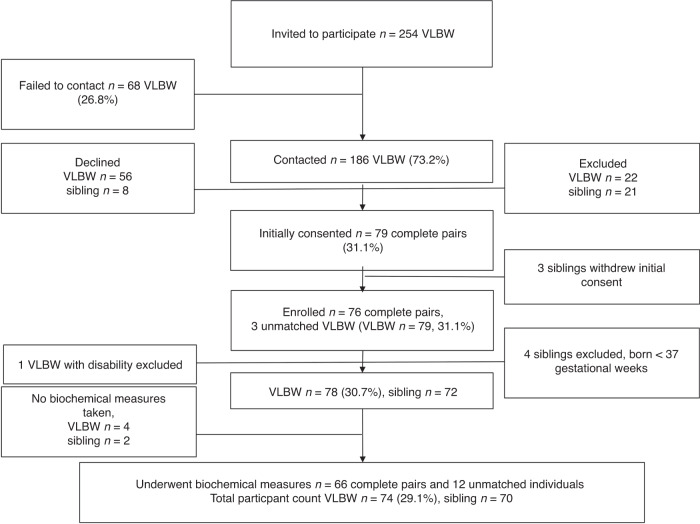
Flowchart of the participants. The proportion of very low birth weight (VLBW) participants included from the original birth cohort are shown in parentheses.

VLBW participants, born between 1978 and 1989, were recruited from three sources: the Helsinki Study of Very Low Birth Weight Adults,^10^ the ESTER Preterm Birth and Early-Life Programming of Adult Health and Disease Study^11^ and via the Finnish Medical Birth Register. The VLBW adults comprise a population-based sample as they were chosen among all newborns in a defined geographic area. The Finnish Medical Birth Register includes data on all live and stillborn neonates in Finland since 1987, with birth weights ≥500 g or gestational age ≥22 weeks. The recruitment (Fig. 1) has been described previously.^15^ In brief, between July 2014 and February 2017, 186 VLBW-adults were contacted, if population records showed that they had a same-sex sibling with less than a 10-year age difference. Each VLBW-adult interested in participating, was asked to inquire whether also their sibling would be willing to participate. All participating siblings were born at term and at least 18 years old. Pregnancy, cerebral palsy, intellectual disability, motor or sensory impairment, severe chronic disease, and endocrine disorders, including type I and II diabetes, were exclusion criteria. As clinical study visits included detailed investigations, with for example magnetic resonance imagining^16,17^ and tissue biopsies, we excluded individuals with manifest diabetes; thus, 1 VLBW and 2 sibling participants were excluded due to type 1 diabetes. Inhaled and topical glucocorticoids were allowed while systemic use was an exclusion criterion. After initial assessment further exclusions were made due to participant related issues (pregnancy, compliance issues, declining further participation) and four controls were found to be born preterm (Fig. 1). All excluded participants have previously been described in detail.^15–18^ Finally, 74 VLBW adults and 70 siblings underwent biochemical measurements (Fig. 1), of these 66 were complete sibling pairs and 12 unmatched participants.

### Ethics

Our study was performed in accordance with the declaration of Helsinki and was approved by the ethics committee of the Hospital District of Helsinki and Uusimaa. All participants provided written informed consent.

Because of individual participant consent, these data are not freely available. Investigators requesting data access should contact the corresponding author (N.K.). Request could be subject to ethics review and/or participant consent.

### Measures and procedures

Perinatal data was collected from maternity clinic and hospital records as previously described.^15^ Based on sex and age, we calculated small for gestational age as birth weight < −2 standard deviations (SDs).^19^

Weight and height were measured by a trained study nurse during the clinical examinations, conducted in 2014–2017. Body mass index (BMI) was calculated as weight divided by height squared (kg/m^2^). All participants completed questionnaires on participant and parental health status, including medical history and medications. Highest parental education served as indicator for childhood socioeconomic status and was categorized into three levels: lower secondary or less, higher secondary and tertiary.

Peripheral venous blood samples were collected and a 2-h, 75 g oral glucose tolerance test (OGTT) was performed after overnight fasting. After drinking the glucose solution, blood samples were drawn at baseline, 30, 60, and 120 min, for analyzing glucose, insulin, and free fatty acids (FFA) levels.

### Laboratory analyses

At the clinical visit, venous blood samples were drawn in a sitting position with a light stasis into a fluoride-citrate tube (Venosafe, Terumo Europa, Leuven, Belgium) for glucose assays and into a tube containing clot activator (Venosafe) for other assays. Fluoride-citrate plasma and serum were separated by centrifuging, frozen locally immediately after separation, and then transported frozen on dry ice to the Biochemistry laboratory at the Finnish Institute for Health and Welfare (Helsinki, Finland). All analyses were performed on a clinical chemistry analyzer (Architect ci8200 Abbott Laboratories, Abbott Park, Illinois). For standardizing measurements, the laboratory has taken part in the Lipid Standardization Program organized by Centers for Disease Control and Prevention (CDC, Atlanta, Georgia) and External Quality Assessment Schemes organized by Labquality (Helsinki, Finland). The between-assay coefficient of variation (CV%, mean ± SD), systematic error (Bias%, mean ± SD) and the principle of the methods in the biochemistry laboratory during the study are shown in Supplementary Table 1.

### Statistical analysis

Power calculations performed before participant recruitment indicated that, with a power of 0.80 and an alpha value of 0.05, two-way paired comparisons within 75 sibpairs would allow us to detect a 0.33 SD difference in continuous outcomes between VLBW adults and sibling-controls. For the actual number of 66 sibling pairs in this paper, the corresponding difference is 0.35 SD. All analyses were performed using IBM SPSS Statistics versions 27 and 28 (IBM Corp. Armonk, NY).

This was a cohort study, and the power of sample size is indicated by confidence intervals. The 95% CI reflects a significance level of 0.05. To estimate variation within each group of data we describe SDs. We used descriptive statistics to illustrate demographic and laboratory test outcomes by group. Background variables were compared using t-test for continuous variables (described using means and SDs) and χ² test for categorical variables (described with counts and percentages). P values were 2-sided and the significance level was set to 0.05.

As laboratory outcomes were not normally distributed, we log-transformed them to attain normality prior to performing statistical analyses. We used linear mixed effects models to assess the effect of VLBW-status on our outcomes, within-sibling analysis compared cardiometabolic biochemical measures. The mixed model incorporates fixed and random effects and is useful for missing values, repeated measurements or when measuring clusters of related statistical units.

We used the following variables as fixed effects: crude model 1 adjusts for age and sex (if applicable), model 2 further adjusts for prenatal and environmental confounders, i.e., maternal gestational or chronic hypertension, preeclampsia, maternal smoking during pregnancy and parental educational attainment, included because of potential effects on both preterm VLBW birth and later offspring health, and model 3 additionally includes participant-related factors: BMI, height, and smoking status, included as possible mediators for the association between preterm VLBW birth and cardiometabolic health.

We used World Health Organization’s (WHO) criteria^20^ for type 2 diabetes (T2D, fasting plasma glucose ≥7.0 mmol/l or 2-h plasma glucose ≥11.1 mmol/l), impaired glucose tolerance (IGT, fasting plasma glucose <7.0 mmol/l and 2-h plasma glucose ≥7.8 mmol/l and <11.1 mmol/l) and impaired fasting glucose (IFG, fasting plasma glucose 6.1 to 6.9 mmol/l and 2-h plasma glucose <7.8 mmol/l). Participants were compared by logistic regression model.

## Results

Study participants are described in Table 1. A total of 144 participants, consisting of 66 complete sibling pairs and 12 unmatched participants, provided blood samples and underwent OGTT. Mean age at participation was 29.2 (SD 3.9) years and 51.4% were women. At adult age, VLBW participants were shorter than their siblings (Table 1). They were also more likely to have been born small for gestational age, with caesarean section and from multiple pregnancy (Table 1).

**Table 1:** Baseline participant characteristics presented by study group: very low birth weight and their controls, i.e., siblings born at term.

Characteristic	VLBW^a^ (*n* = 74)	Term-born sibling (*n* = 70)
Birth/Perinatal characteristics		
Birth weight, mean (SD), g	1160 (215)	3404 (432)*
Birth weight SD score, mean (SD)	−1.4 (1.6)	−0.3 (0.9)*
Gestational age, mean (SD), weeks	29.6 (2.4)	39.8 (1.3)*
Male, *n* (%)	36 (48.6)	34 (48.6)
Small for gestational age, *n* (%)	28 (37.8)	2 (2.9)*
Caesarean section, *n* (%)	31 (41.9)	12 (16.7)**
Maternal smoking during pregnancy, *n* (%)	11 (14.9)	11 (15.7)
Multiple pregnancy, *n* (%)	7 (9.5)	1 (1.4)***
Gestational and chronic hypertension, *n* (%)	4 (5.4)	18 (25.7)**
Pre-eclampsia (PE) and superimposed PE *n* (%)	20 (27.0)	1 (1.4)*
Proteinuria, *n* (%)	2 (2.7)	5 (7.1)
Firstborn, *n* (%)	29 (39.2)	23 (32.9)
Current characteristics		
Age, mean (SD), years	29.4 (2.6)	29.0 (4.9)
Smoker, *n* (%)	18 (24.3)	23 (32.9)
BMI, mean (SD), kg/m^2^		
Men	25.0 (3.8)	25.3 (3.9)
Women	24.2 (5.3)	23.7 (5.0)
BMI ≥ 25 kg/m^2^, *n* (%)	26 (35.1)	23 (32.9)
BMI ≥ 30 kg/m^2^, *n* (%)	10 (13.5)	9 (12.9)
Height, mean (SD), cm		
Men	173.8 (8.1)	180.1 (6.9)***
Women	161.1 (6.4)	165.3 (5.6)**
Parental education, *n* (%)		
Lower secondary or less	0	0
Higher secondary	29 (39.2)	25 (35.7)
Tertiary	44 (59.5)	44 (62.9)
Maternal medical conditions at offspring mean age 29 years, *n* (%)^b^		
Hypertension	21 (28.4)	18 (25.7)
Diabetes	4 (5.4)	3 (4.3)
Stroke or myocardial infarction	4 (5.4)	4 (5.7)
Paternal medical conditions at offspring mean age 29 years, *n* (%)^b^		
Hypertension	20 (27.0)	14 (20.0)***
Diabetes	14 (18.9)	9 (12.9)
Stroke or myocardial infarction	9 (12.2)	5 (7.1)

*T*-test for continuous and χ2 test for categorical variables.

*Denotes significant difference of *p* < 0.001; **Denotes significant difference of *p* < 0.01; ***Denotes significant difference of *p*  < 0.05.

^a^Very low birth weight, <1500 g.

^b^As reported by the participant.

As our sample size is limited, we included all VLBW-individuals in the same group, both participants born small for gestational age (SGA: men *n* = 10, women *n* = 18) and appropriate for gestational age (AGA: men *n* = 26, women *n* = 20). However, we reran the analyses separately comparing VLBW-SGA with controls and VLBW-AGA with controls and present the results for additional information (Supplementary Table 2). This data should be interpreted with caution as our study is not powered to detect or exclude such associations, especially in analyses stratified by sex.

### Cardiometabolic markers

Means of cardiometabolic biochemical measures for VLBW and sibling participants are shown in Table 2 and comparisons between groups using linear mixed models are presented in Table 3.

**Table 2:** Cardiometabolic biomarkers of young adults born at very low birth weight and their controls, siblings born at term.

Characteristic	VLBW^a^ (*n* = 74)	Term-born sibling (*n* = 70)
	mean (SD)	mean (SD)
Fasting glucose, mmol/l	5.4 (0.4)	5.3 (0.4)
30 min glucose, mmol/l	8.6 (1.7)	8.8 (1.4)
60 min glucose, mmol/l	8.6 (2.1)	8.4 (2.1)
120 min glucose, mmol/l	7.0 (1.8)	6.4 (1.6)*
Fasting insulin, mU/l	6.9 (3.7)	7.3 (4.4)
30 min insulin, mU/l	55.7 (32.5)	59.6 (35.3)
60 min insulin, mU/l	52.2 (31.3)	58.1 (41.4)
120 min insulin, mU/l	47.2 (43.0)	44.4 (39.4)
Fasting free fatty acids, mmol/l	0.64 (0.25)	0.57 (0.26)
30 min free fatty acids, mmol/l	0.30 (0.14)	0.32 (0.13)
60 min free fatty acids, mmol/l	0.12 (0.06)	0.11 (0.05)
120 min free fatty acids, mmol/l	0.05 (0.03)	0.05 (0.05)
Total cholesterol, mmol/l	4.7 (1.3)	4.6 (0.8)
HDL cholesterol, mmol/l		
Men	1.3 (0.3)	1.3 (0.3)
Women	1.6 (0.4)	1.6 (0.4)
LDL cholesterol, mmol/l	2.8 (1.2)	2.7 (0.7)
Triglycerides, mmol/l	1.0 (0.6)	0.9 (0.4)
Homocysteine, μmol/l	2.0 (0.3)	2.0 (0.3)
Apolipoprotein A1, g/l		
Men	1.5 (0.2)	1.5 (0.2)
Women	1.7 (0.3)	1.7 (0.3)
Apolipoprotein B g/l		
Men	0.9 (0.4)	0.9 (0.2)
Women	0.8 (0.2)	0.8 (0.1)
Sex hormone binding globulin nmol/l		
Men	35.8 (16.6)	32.7 (12.1)
Women	106.8 (82.9)	92.6 (69.5)
Testosterone, nmol/l		
Men	21.3 (6.7)	20.3 (5.8)
Women	1.2 (0.5)	1.1 (0.6)
Uric acid, μmol/l	311.8 (79.0)	300.2 (65.7)
Ferritin, μg/l	71.3 (55.4)	82.2 (73.2)
High-sensitivity C-reactive protein^b^, mg/l	1.5 (1.7)	1.5 (2.0)
Complement component C3, g/l	1.1 (0.2)	1.1 (0.2)
Complement component C4, g/l	0.2 (0.1)	0.2 (0.1)
Alanine aminotransferase, U/l	20.3 (14.0)	20.3 (13.1)
Aspartate transaminase, U/l	25.5 (8.9)	25.4 (6.8)
Alkaline phosphatase, U/l	66.0 (18.4)	68.2 (20.4)

All results are presented as arithmetic means.

*Denotes significant difference of *p* < 0.05.

^a^Very low birth weight <1500 g.

^b^4 participants had a high-sensitivity C-reactive protein >10 mg/l, these were excluded from analyses.

**Table 3:** Comparison of cardiometabolic biomarkers between young adults born at very low birth weight and their sibling-controls.

	Term born sibling (*n* = 70)	VLBW^a^ (*n* = 74) vs siblings	
Characteristic or measure	Geometric mean (SD)	Mean difference, %^b^	95 % Confidence interval, %
Fasting glucose, mmol/l	5.3 (1.1)		
Model 1		0.7	−1.5, 2.9
Model 2		0.6	−1.7, 2.9
Model 3		1.7	−0.9, 4.3
30 min glucose, mmol/l	8.7 (1.2)		
Model 1		−2.8	−8.3, 3.1
Model 2		−1.5	−7.8, 5.1
Model 3		−4.2	−10.9, 3.2
60 min glucose, mmol/l	8.2 (1.3)		
Model 1		1.4	−7.1, 10.7
Model 2		4.2	−5.4, 14.8
Model 3		5.5	−5.6, 18.0
120 min glucose, mmol/l	6.2 (1.3)		
Model 1		9.2	0.4, 18.8*
Model 2		11.3	2.4, 21.0*
Model 3		10.3	0.2, 21.3*
Fasting insulin, mU/l	6.6 (1.5)		
Model 1		−6.3	−18.3, 7.5
Model 2		−6.6	−19.5, 8.4
Model 3		0.0	−13.5, 15.5
30 min insulin, mU/l	49.8 (1.9)		
Model 1		−7.3	−24.3, 13.5
Model 2		−6.5	−25.2, 16.9
Model 3		−11.3	−28.9, 10.5
60 min insulin, mU/l	46.9 (1.9)		
Model 1		−4.5	−22.3, 17.3
Model 2		−4.3	−23.1, 19.1
Model 3		8.2	−15.1, 37.9
120 min insulin, mU/l	32.3 (2.3)		
Model 1		5.3	−17.8, 34.9
Model 2		10.9	−15.6, 45.9
Model 3		10.2	−17.9, 47.9
Fasting free fatty acids, mmol/l	0.51 (1.65)		
Model 1		13.4	−0.3, 29.0
Model 2		17.4	3.4, 33.4*
Model 3		19.0	2.7, 37.9*
30 min free fatty acids, mmol/l	0.29 (1.62)		
Model 1		−6.9	−21.4, 10.2
Model 2		−3.7	−19.0, 14.8
Model 3		−2.9	−19.6, 17.2
60 min free fatty acids, mmol/l	0.10 (1.59)		
Model 1		5.2	−11.8, 25.4
Model 2		7.8	−10.3, 29.5
Model 3		12.3	−7.5, 36.3
120 min free fatty acids, mmol/l	0.04 (1.84)		
Model 1		15.6	−2.4, 36.9
Model 2		16.4	−3.1, 39.9
Model 3		29.7	7.4, 56.7**
Testosterone in men, nmol/l	20.3 (1.4)		
Model 1		3.5	−8.6, 17.1
Model 2		3.6	−9.8, 18.9
Model 3		2.5	−10.2, 17.1
Testosterone in women, nmol/l	1.1 (1.6)		
Model 1		9.1	−7.5, 28.8
Model 2		10.6	−5.3, 29.1
Model 3		15.1	−3.3, 37.1
Serum sex hormone binding globulin in men, nmol/l	19.5 (1.3)		
Model 1		6.1	−10.7, 26.0
Model 2		7.5	−9.3, 27.4
Model 3		6.8	−11.3, 28.7
Sex hormone binding globulin in women, nmol/l	77.5 (1.8)		
Model 1		4.7	−18.0, 33.6
Model 2		1.3	−22.6, 32.5
Model 3		3.5	−23.0, 39.2
Total cholesterol, mmol/l	4.6 (1.2)		
Model 1		0.4	−5.4, 6.5
Model 2		0.2	−5.9, 6.7
Model 3		−0.9	−7.5, 6.0
HDL cholesterol in men, mmol/l	1.3 (1.3)		
Model 1		−3.9	−12.7, 5.9
Model 2		−1.3	−10.2, 8.5
Model 3		1.8	−8.6, 13.3
HDL cholesterol in women, mmol/l			
Model 1	1.5 (1.3)	3.3	−5.0, 12.4
Model 2		2.5	−6.4, 12.3
Model 3		1.6	−6.6, 10.4
LDL cholesterol, mmol/l	2.7 (1.3)		
Model 1		−0.5	−8.7, 8.4
Model 2		−0.9	−9.7, 8.7
Model 3		−3.5	−12.6, 6.4
Triglycerides, mmol/l	0.9 (1.5)		
Model 1		2.7	−8.1, 14.8
Model 2		1.7	−9.8, 14.8
Model 3		10.5	−1.7, 24.3
Homocysteine, μmol/l	2.0 (1.2)		
Model 1		0.5	−3.2, 4.3
Model 2		0.9	−3.0, 4.8
Model 3		2.4	−1.8, 6.8
Apolipoprotein A1 in men, g/l	1.5 (1.2)		
Model 1		−0.8	−7.4, 6.3
Model 2		0.7	−6.1, 7.9
Model 3		4.5	−3.4, 13.1
Apolipoprotein A1 in women, g/l	1.7 (1.2)		
Model 1		2.1	−3.5, 7.9
Model 2		1.6	−4.5, 8.0
Model 3		2.1	−3.8, 8.3
Apolipoprotein B in men, g/l	0.7 (1.2)		
Model 1		−2.9	−14.1, 9.6
Model 2		−3.2	−14.8, 9,8
Model 3		−2.4	−14.7, 11,7
Apolipoprotein B in women, g/l	0.7 (1.2)		
Model 1		1.1	−7.0, 9.9
Model 2		1.7	−7.0, 11.1
Model 3		1.4	−7.5, 11.0
Uric acid, μmol/l	292.9 (1.3)		
Model 1		3.6	−1.6, 9.0
Model 2		4.3	−1.0, 10.0
Model 3		5.6	−0.7, 12.3
Ferritin, μg/l	57.6 (2.6)		
Model 1		−16.2	−33.0, 4.7
Model 2		−17.1	−35.0, 5.9
Model 3		−18.8	−38.5, 7.1
High-sensitivity C-reactive protein, mg/l	0.8 (2.9)		
Model 1		−0.8	−29.9, 40.6
Model 2		−1.5	−31.0, 40.6
Model 3		6.9	−24.6, 51.6
Complement component C3, g/l	1.1 (1.2)		
Model 1		−0.5	−6.1, 5.4
Model 2		−1.5	−7.2, 4.6
Model 3		−0.7	−6.3, 5.3
Complement component C4, g/l	0.2 (1.3)		
Model 1		−5.4	−12.1, 1.9
Model 2		−5.7	−12.5, 1.8
Model 3		−3.6	−10.9, 4.3
Alanine aminotransferase, U/l	17.4 (1.7)		
Model 1		−2.2	−14.5, 11.8
Model 2		−1.2	−14.4, 14.1
Model 3		−0.6	−14.9, 16.1
Aspartate transaminase, U/l	24.6 (1.3)		
Model 1		−1.2	−7.9, 6.1
Model 2		0.1	−6.9, 7.6
Model 3		0.1	−8.1, 9.0
Alkaline phosphatase, U/l	65.4 (1.3)		
Model 1		−2.3	−10.1, 6.1
Model 2		−2.0	−10.3, 6.9
Model 3		−2.3	−11.7, 8.0

Mixed models are as follows:

Model 1, adjusted for age and sex (if applicable).

Model 2, adjusted for age, sex (if applicable), maternal hypertension or preeclampsia during pregnancy, maternal smoking during pregnancy and parental educational attainment.

Model 3, adjusted for age, sex (if applicable), maternal hypertension or preeclampsia during pregnancy, maternal smoking during pregnancy, parental educational attainment, BMI, height and smoking status.

*Denotes significant difference of *p*  < 0.05; **Denotes significant difference of *p* < 0.01.

^a^Very low birth weight <1500 g.

^b^Statistical comparisons with mixed models are presented as geometric means, corresponding to % difference.

In the OGTT (Tables 3), 2-h glucose concentrations were higher (mean difference 9.2%, 95% CI [0.4, 18.8], *P* = 0.04) in VLBW participants as compared with siblings, while no differences were found in fasting glucose, fasting insulin or 2-h insulin concentrations. Both fasting FFA and 2-h FFA were higher in VLBW-adults (models 1–3).

**Fig. 2:**
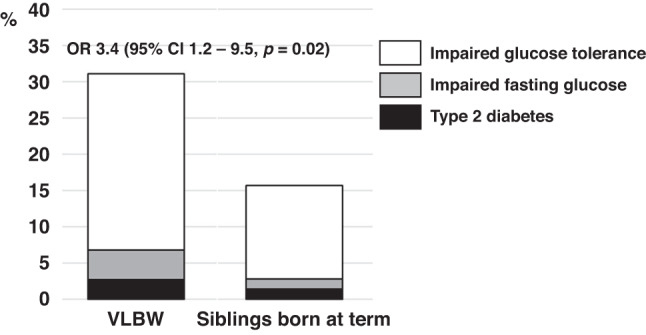
Impaired glucose regulation in adults born preterm at very low birth weight (VLBW) and term-born siblings. The Odds ratio (OR) is adjusted for age, sex, maternal smoking, hypertension and preeclampsia during pregnancy, parental educational attainment, body mass index, height and smoking status.

2 VLBW and 1 sibling met the WHO criteria^20^ for T2D, while 3 VLBW and 1 sibling had IFG, and IGT was found in 18 VLBW and 9 siblings (Table 4). When any form of impaired glucose regulation, i.e., T2D, IFG, or IGT was studied as an outcome (Fig. 2), VLBW-adults showed a 2.5- to 3.4-fold increase compared with sibling participants (Table 4).

**Table 4:** Impaired glucose regulation in young adults born at very low birth weight and their sibling-controls, born at term. Comparisons are presented by study group.

Characteristic	VLBW^a^ (*n* = 74)	Term-born sibling (*n* = 70)
T2D^b^, *n* (%)	2 (2.7)	1 (1.4)^c^
Impaired glucose tolerance^b^, *n* (%)	18 (24.3)	9 (12.9)^c^
Impaired fasting glucose^b^, *n* (%)	3 (4.1)	1 (1.4)^c^
Impaired glucose regulation^d^, *n* (%)	23 (31.1)	11 (15.7)^c^*
Odds ratio for impaired glucose regulation^d^		
Model 1, 95% confidence interval	2.5	1.1, 5.8*
Model 2, 95% confidence interval	3.0	1.2, 7.4*
Model 3, 95% confidence interval	3.4	1.2, 9.5*

Model 1, adjusted for age and sex;

Model 2, adjusted for age, sex, maternal hypertension or preeclampsia during pregnancy, maternal smoking during pregnancy and parental educational attainment;

Model 3, adjusted for age, sex, maternal hypertension or preeclampsia during pregnancy, maternal smoking during pregnancy, parental educational attainment, BMI, height and smoking status.

*Denotes significant difference of *p* < 0.05.

^a^Very low birth weight < 1500 g.

^b^According to World Health Organization’s definition.

^c^χ² test.

^d^Includes T2D, impaired glucose tolerance and impaired fasting glucose.

Regarding other cardiometabolic biomarkers, including testosterone, sex hormone binding globulin, lipid profiles, uric acid, ferritin, inflammatory markers and liver tests, findings were similar between groups (Tables 2 and 3). 3 VLBW and 1 sibling participant had a high-sensitivity C-reactive protein greater than 10 mg/l and were excluded from high-sensitivity C-reactive protein and ferritin analyses.

### Cardiometabolic biomarkers in comparisons between complete sibling pairs

After excluding 12 unmatched participants, we reran all analyses to separately compare complete sibling pairs. Most results remained similar. However, in the OGTT, the difference in 2-h glucose was somewhat attenuated: model 1 mean difference 8.3% (95% CI [−0.9, 18.2], *P* = 0.08), model 2 10.7% (95% CI [1.5, 20.7], *P* = 0.02), and model 3 9.0% (95% CI [−1.1, 20.2], *P* = 0.08).

In addition, compared with siblings, testosterone was 15.0–18.3% higher in VLBW-women, reaching statistical significance only in the fully adjusted model 3 (mean difference 18.3%, 95% CI [0.3, 39.6], *P* = 0.05). In men, no such difference was found.

## Discussion

This cohort study compared cardiometabolic biomarkers in young adults born preterm at VLBW with their same-sex, term-born siblings. VLBW-adults had 3.4-fold odds for impaired glucose regulation compared with their siblings. They also showed higher 2-h glucose concentrations, fasting and 2-h FFA concentrations. No differences were, however, seen in fasting glucose, fasting insulin or 2-h insulin concentrations. Other cardiometabolic biomarkers, including lipid profiles, liver tests, inflammatory markers, and measures of hyperandrogenism were similar between groups.

Previous studies with unrelated controls have shown robust associations between preterm birth and increased risk for later chronic diseases, including cardiovascular disease, metabolic syndrome and diabetes.^8,21^ In a Swedish cohort study, involving 674820 participants, each week of increased prematurity resulted in a 7% higher risk of dying in young adulthood from cardiovascular disease.^22^ Another study, including one of our source cohorts, comparing VLBW with unrelated, term-born controls,^10^ showed 5.3% higher 2-h glucose, 12.6% higher fasting insulin and 32.8% higher 2-h-insulin concentrations in VLBW individuals in their mid-twenties. In our study insulin concentrations were similar between VLBW and sibling participants, while earlier studies, including two meta-analyses, have suggested point estimates ranging from 8% to 18.7%,^8,10,23^ which is beyond our 95% confidence interval for fasting insulin (−18.3%, 7.5%). However, the point estimate of 2-h insulin, 32.8%, in the study with unrelated controls,^10^ was included in the confidence intervals (−17.8%, 34.9%) of our study, although it came close to the upper limits. This suggests that shared family factors may partially explain differences in insulin sensitivity between VLBW adults and full-term controls.

By contrast to lack of difference in insulin sensitivity between groups in the current study, our finding of 9.2% higher 2-h glucose concentrations in VLBW-participants, corresponds to the previously reported difference of 5.3% in the source cohort study with unrelated controls^10^ and approximately 1.4–2.4% in two meta-analyses^8,23^ that included also non-VLBW preterm-born adults. This points at susceptibility for impaired glucose metabolism and increased risk of diabetes later in life in adults born preterm at VLBW. Our participants were relatively young, close to 30 years of age. It is probable that the clinical implications of VLBW regarding cardiometabolic disorders will mostly become apparent at a later age. While only relatively few study participants fulfilled the WHO criteria^20^ for T2D or IFG, risk of any category of impaired glucose regulation was 3.4-fold in VLBW-participants compared with siblings (Fig. 2). This is only slightly lower than the 4.0-fold risk in a cohort study of extremely low birth weight adults utilizing matched, normal birth weight controls.^24^ Similarly, in a recent study by Flahault et al., at mean age 23 years, glucose intolerance was 2.2-fold more common in former very preterm born (≤29 weeks) compared to full-term controls.^25^ That study attempted to mitigate bias caused by unmeasured confounding factors regarding environment and lifestyle, by choosing relatives and friends of the preterm participants as controls.

While not directly comparable to our study, Darlow et al.^26^ found that VLBW adults, aged 26–30, displayed poorer physiological functioning than term-born controls. This was done by comparing an aggregate score consisting of 10 physiological biomarkers, such as blood pressure and cholesterol. Although preterm birth is considered a risk factor for metabolic syndrome and cardiovascular disease in adult life,^8^ we found no evidence on more atherogenic lipid profiles, signs of hyperandrogenism, elevated liver tests or inflammatory markers in VLBW participants.

In our study, the largest group with impaired glucose regulation (Fig. 2) was the one with IGT, which is characterized by insulin secretion inadequate for the glucose load.^20^ Insulin secretion plays a central role in the development of T2D as most risk genes for T2D influence insulin secretion and not insulin resistance, and FFAs are known to influence insulin secretion.^27^ We did find higher fasting and 2-h FFA concentrations after a 75 g glucose dose in VLBW vs sibling participants. FFAs play a central role in the body’s energy production. Fat is stored in adipose tissue as triglycerides, and FFAs are formed when lipolysis breaks down triglycerides, a process inhibited by insulin. FFAs cause insulin resistance and inflammation, linking obesity, insulin resistance, inflammation, T2D, dyslipidemia and atherosclerotic disease.^28^ Our findings of higher fasting and 2-h FFA concentrations in VLBW participants, might be a sign of insulin resistance of adipose tissue,^29^ i.e., insulin is not suppressing FFA formation (lipolysis) after a glucose load. Higher fasting and 2-h FFA concentrations in VLBW participants could also indicate that prematurity influences energy metabolism. Another indication of differences in metabolism was previously reported in one of our source cohorts, in which VLBW-adults had a higher than expected resting energy expenditure based on lean body mass,^30^ suggesting the presence of more metabolically active tissue in VLBW individuals. Likewise, recently published data from our sibling study cohort, showed less unsaturation in subcutaneous adipose tissue in VLBW adults,^16^ also suggesting differences in fat metabolism.

Traditionally, in studies on long-term effects of preterm birth, the control groups constitute of healthy term-born individuals. In our study, the lack of difference between VLBW and sibling controls regarding several common risk factors of cardiovascular disease might be explained by shared genetic, socioeconomic, or environmental factors in the families. Our findings support this, the differences between groups in cardiometabolic risk factors seem smaller than in studies utilizing full-term, unrelated study participants as controls. For instance, compared to our study, a previous non-sibling cohort study reported larger differences in alanine aminotransferase levels (15.0% vs −2.2%), aspartate transaminase levels (11.7% vs −1.2%) and uric acid levels (20.1% vs 3.6%), with point estimates of that study not even included in our study’s confidence intervals.^11^ This suggests that shared family factors may partly explain previously reported differences in these biomarkers between VLBW adults and controls born at term. Further, the part not explained by shared family factors or pregnancy-related confounders could be explained by postnatal environment exposures encountered after VLBW birth.

Previous, non-sibling cohort studies on effects of preterm birth on cardiometabolic health also report differences regarding total cholesterol, low-density lipoprotein cholesterol and apolipoprotein B concentrations^31^ although this is not found consistently in all studies.^8,10,23^ Similarly, in large systematic reviews and meta-analyses preterm born adults have exhibited higher total cholesterol^8^ and low-density lipoprotein cholesterol levels,^23^ while in our study, the atherogenic lipid profiles did not differ between VLBW and siblings groups.

Our sibling design has inherent strengths and limitations. The most important strength is to circumvent many unmeasured familial confounders. Furthermore, we adjusted for many confounders that vary within siblings, including maternal, pregnancy-related factors and current participant related mediators. The most important limitation is that we recruited VLBW participants with an obliging same-sex sibling. As the protocol was extensive, there might be a bias towards sibling pairs who are more similar and closely connected, which could lead to more conservative findings. It is also possible that childhood environmental exposures and family structure between siblings vary, as the maximum allowed age difference between participants was 10 years, which we were not able to account for. Because our sample size is limited, we included all VLBW participants, both SGA and AGA, in the same group. Although we present these results to the readers (Supplementary Table 2), results on SGA and AGA should be treated with caution as this study is not powered to detect or exclude such associations, especially in analyses stratified by sex. Further, to make good use of all data collected, we included 12 unmatched participants in the analyses. In addition, our study population is Finnish, and the results may therefore not be directly extrapolated globally to people with different genetic backgrounds.

## Conclusion

Preterm birth at VLBW is a risk factor for impaired glucose metabolism. Already as young adults the VLBW participants of our cohort displayed signs of IGT. In contrast to previous studies, differences in insulin resistance were not apparent, suggesting that insulin resistance may partially be explained by factors shared between siblings. Further, common cardiometabolic biomarkers, including lipid profiles, signs of hyperandrogenism, liver tests and inflammatory markers were similar within sibling pairs.

Considering that 10% of the population is born preterm, the impact of perinatal history is relevant and significantly effects life-long public health.

### Supplementary information

Supplementary Tables

## Data Availability

Deidentified individual participant data is not publicly available. Because of individual participant consent these data are not freely available. Investigators requesting data access should contact the corresponding author (N.K.). Request could be subject to ethics review and/or participant consent.
