# Supplementary material for: Cardiometabolic health in adults born with very low birth weight—a sibling study
Source: Pediatr Res. 2023 Sep 27;95(1):316–24. doi: 10.1038/s41390-023-02828-3 (PMC10798885; doi:10.1038/s41390-023-02828-3)
Supplement: Supplementary file 1 — Supplementary Tables [file 41390_2023_2828_MOESM1_ESM.pdf]

Supplementary Table 1. Biochemistry laboratory methods used, with the between-assay coefficients of variation (CV%, mean ±SD) and systematic errors (Bias%, mean ±SD) according to the External Quality Assessment Schemes shown.

Assay	Method	Reagents	CV% (N[a]),mean ±SD, (Study)	BIAS% (N[b]), mean ±SD, (Study)
Alanine amino transferase	Photometric, kinetic (IFCC)	Abbott Laboratories	(7) 3.9% ±2.6	(11) 3.0% ±4.6
Alkaline phosphatase	Photometric, kinetic (IFCC)	Abbott Laboratories	(2) 0.7%±0.3	(2) 1.8%±1.3
Apolipoprotein A1	Immunoturbidimetric	Abbott Laboratories	(5) 1.4% ±1.0	(10) 3.0% ±3.9
Apolipoprotein B	Immunoturbidimetric	Abbott Laboratories	(5) 1.9% ±3.0	(10) 3.3% ±4.0
Aspartate aminotransferase	Photometric, kinetic (IFCC)	Abbott Laboratories	(7) 0.6% ±0.4	(11) 4.3% ±2.9
Complement C3	Immunoturbidimetric	Abbott Laboratories	(3) 1.0%±0.4	
Complement C4	Immunoturbidimetric	Abbott Laboratories	(3) 1.5%±1.1	
Cholesterol, total	Enzymatic	Abbott Laboratories	(5) 0.7% ±0.2	(30) -0.3% ±0.8
Cholesterol, HDL	Enzymatic, homogenous direct	Abbott Laboratories	(5) 1.0% ±0.6	(10) -0.6% ±1.0
Cholesterol, LDL	Calculated by Friedewald			
C-Reactive protein (high sensitivity)	Immunoturbidimetric	Abbott Laboratories	(3) 3.4% ±1.0	(8) -3.6% ±5.6
Ferritin	Chemiluminescent Microparticle Immuno Assay	Abbott Laboratories	(2) 2.0% ±1.5	(1) -0.4%

Free fatty acids	Enzymatic	Abbott Laboratories	(3) 2.0% ±0.3	
Glucose	Enzymatic, hexokinase	Abbott Laboratories	(5) 1.4% ±0.9	(10) 4.5% ±2.3
Homocysteine	Chemiluminescent Microparticle Immuno Assay	Abbott Laboratories	(2) 1.1% ±0.2	
Insulin	Chemiluminescent Microparticle Immuno Assay	Abbott Laboratories	(4) 4.9% ±3.2	(2) -0.1% ±2.4
Sex-hormone binding globulin	Chemiluminescent Microparticle Immuno Assay	Abbott Laboratories	(2) 4.0% ±5.3	
Testosterone	Chemiluminescent Microparticle Immuno Assay	Abbott Laboratories	(2) 3.5% ±1.6	
Triglycerides	Enzymatic	Abbott Laboratories	(5) 1.5% ±1.5	(10) -1.1% ±4.2
Uric Acid	Enzymatic	Abbott Laboratories	(2) 0.4% ±0.2	(1) -0.6%

[a]Number of different quality control samples
[b]Number of reference samples

Supplementary Table 2. Comparison of cardiometabolic biomarkers between young adults born at very low birth weight and their sibling-controls. The results are presented separately for small for gestational age VLBW and appropriate for gestational age VLBW participants.

	**SGA VLBW[a] (n=28) vs. siblings (n=70)**		**AGA VLBW[a] (n=46) vs. siblings (n=70)**	
Characteristic or measure	Mean difference, %[b]	95 % Confidence interval, %	Mean difference, %[b]	95 % Confidence interval, %
Fasting glucose, mmol/l	-0.4	-3.7, 3.0	1.1	-1.3, 3.6
30 min glucose, mmol/l	-2.1	-9.9, 6.4	-3.6	-9.7, 3.0
60 min glucose, mmol/l	5.2	-7.3, 19.4	-1.2	-10.8, 9.3
120 min glucose, mmol/l	13.2	1.0, 26.9†	6.0	-3.9, 17.0
Fasting insulin, mU/l	-9.1	-24.9, 10.1	-4.6	-18.1, 11.1
30 min insulin, mU/l	-9.0	-32.7, 22.9	-8.8	-25.5, 11.6
60 min insulin, mU/l	-2.8	-28.8, 32.8	-8.5	-27.9, 16.2
120 min insulin, mU/l	13.2	-21.6, 63.5	1.7	-23.1, 34.5
Fasting free fatty acids, mmol/l	24.0	4.7, 46.9†	7.3	-8.4, 25.8
30 min free fatty acids, mmol/l	-2.1	-22.9, 24.3	-10.3	-25.6, 8.2
60 min free fatty acids, mmol/l	7.7	-14.5, 35.7	1.9	-17.1, 25.4
120 min free fatty acids, mmol/l	25.4	1.6, 54.8†	10.7	-10.5, 36.8

Testosterone in men, nmol/l	2.5	-17.5, 27.2	5.3	-7.0, 19.3
Testosterone in women, nmol/l	14.9	-6.9, 41.7	4.4	-16.1, 29.9
Serum sex hormone binding globulin in men, nmol/l	-6.1	-28.7, 23.7	11.8	-3.8, 29.9
Sex hormone binding globulin in women, nmol/l	4.4	-25.3, 45.9	5.7	-21.0, 41.6
Total cholesterol, mmol/l	5.5	-3.1, 14.9	-3.0	-8.4, 2.7
HDL cholesterol in men, mmol/l	-9.3	-22.2, 5.7	-1.8	-11.8, 9.2
HDL cholesterol in women, mmol/l	2.2	-8.4, 14.0	5.1	-6.7, 18.3
LDL cholesterol, mmol/l	7.7	-4.5, 21.4	-5.4	-13.3, 3.2
Triglycerides, mmol/l	*12.5*	*-4.8, 33.0*	*-2.6*	-14.0, 10.3
Homocysteine, µmol/l	-0.7	-6.2, 5.1	0.9	-3.4, 5.5
Apolipoprotein A1 in men, g/l	-3.1	-12.5, 7.3	-0.2	-7.8, 8.0
Apolipoprotein A1 in women, g/l	3.5	-3.0, 10.5	1.6	-6.0, 9.9
Apolipoprotein B in men, g/l	11.5	-8.3, 35.5	-8.3	-17.4, 1.8
Apolipoprotein B in women, g/l	8.3	0.2, 17.1†	-2.3	-12.8, 9.6
Uric acid, µmol/l	6.8	0.6, 13.4†	1.4	-4.9, 8.2
Ferritin, µg/l	-17.5	-38.0, 9.8	-16.6	-36.2, 9.1
High-sensitivity C-reactive protein, mg/l	29.4	-20.6, 110.6	-13.9	-40.4, 24.4
Complement component C3, g/l	3.0	-5.2, 11.9	-2.4	-8.2, 3.6

Complement component C4, g/l	-1.2	-10.8, 9.5	-7.6	-15.1, 0.7
Alanine aminotransferase, U/l	0.7	-18.0, 23.6	-3.8	-17.4, 12.2
Aspartate transaminase, U/l	3.2	-7.6, 15.3	-3.9	-10.5, 3.1
Alkaline phosphatase, U/l	0.9	-11.3, 14.7	-4.6	-12.6, 4.1

[a] Very low birth weight <1500g

[b] Statistical comparisons with mixed model, adjusted for age and sex (if applicable), are presented as geometric means, corresponding to % difference.

†Denotes significant difference of $p < 0.05$.
